# Coherence time limit of the biphotons generated in a dense cold atom cloud

**DOI:** 10.1038/srep09126

**Published:** 2015-03-17

**Authors:** Zhiguang Han, Peng Qian, L. Zhou, J. F. Chen, Weiping Zhang

**Affiliations:** 1 Quantum Institute for Light and Atoms, State Key Laboratory of Precision Spectroscopy, Department of Physics, East China Normal University, No. 500 Dongchuan Road, Shanghai, 200241, China

## Abstract

Biphotons with narrow bandwidth and long coherence time can enhance light-atom interaction, which leads to strong coupling between photonic and atomic qubits. Such strong coupling is desirable in quantum information processing, quantum storage and communication. In particular, paired photons with a long coherence time over submicroseconds facilitate the direct manipulation of biphoton wavefunction. In this paper, we report the narrow-band biphotons with a coherence time of 2.34 *μ*s generated from spontaneous four-wave mixing (SFWM) in a dense cold atom cloud, in which the anti-Stokes photons go through a narrow electromagnetically-induced transparency (EIT) window. In our knowledge, this is the best record of coherence time for paired photons achieved so far. A number of factors limiting the coherence time are analyzed in detail. We find the EIT coherence plays an essential role in determining the coherence time for paired photons. The EIT dephasing rate is the ultimate limit to the coherence time, and an ultra-long coherence time above ten microseconds is possible by further improvement of the dephasing rate below 100 kHz.

Nonclassical paired photons play an important role in quantum information processing, quantum storage and telecommunication. They are highly correlated in pairs and their second-order cross correlation function violates the Cauchy-Schwartz inequality. These paired photons constitute heralded single photon source, i.e., when one of them is detected, its counterpart can be considered as a single photon in Fock state. Traditional schemes, spontaneous parametric down conversion (SPDC) in nonlinear crystal, generate broadband paired photons with linewidth up to the order of THz^1^,^2^,^3^,^4^,^5^. The paired photons produced in the SPDC scheme are usually considered to be simultaneous, with their coherent time separation, i.e., coherence time, of the order of picosecond. However, efficient quantum state transfer at light-atom interface requires narrow-band paired photon source or narrow-band single photons. SPDC with high finesse cavity^6^,^7^,^8^,^9^ efficiently generates paired photons with linewidths around 10 MHz, and further suppression below atomic natural linewidth (e.g., 6 MHz for rubidium atoms) demands delicate cavity fabrication^10^. Write-read sequenced four-wave mixing process provide an access to the generation of narrow-band paired photons in atomic gas^11^,^12^, and the paired photons produced through atomic spin states storing and retrieving are shown to be highly correlated^13^. Instead of time-separate writing and reading processes, spontaneous four-wave mixing (SFWM) with both controlling beams present simultaneously in a generation cycle is utilized to produce time-frequency entangled paired photons, i.e., biphotons, in cold atomic ensembles^14^,^15^,^16^. Therefore in time-frequency domain, the two-photon state can be expressed by a temporal correlation function, or a probability wavefunction^17^,^18^.

The coherence time of the biphotons is defined as the temporal length of the biphoton correlation function, and it represents the largest time distance of the biphotons emitted from a source. SPDC scheme produce wide-band biphotons with the temporal wavefunction too short to be detected directly by the fastest single photon counter, or to be modulated directly in time domain. Moreover, the ultra-short coherence time complicates the future quantum networking based on the entangled paired photons emitted from independent sources, which require accurate synchronization of the excitation laser pulses^19^,^20^. To largely increase the biphoton coherence time, in this report, we demonstrate the generation of biphotons from a cloud of laser cooled ^85^Rb atoms prepared in a two-dimensional magneto-optical trap (MOT), with the coherence time exceeding far above nanoseconds. When electromagnetically induced transparency (EIT) is introduced into the SFWM process, the linewidth and corresponding temporal length of the biphoton correlation function can be manipulated by controlling the slow light effect of the produced photons. More interestingly, if one could produce a single photon with temporal waveform up to tens of *μ*s, demanding 10 kHz modulation bandwidth, the single photon source could then be easily manipulated as it is an optical pulse. These ultranarrow-band paired photons have shown great advantage in heralded single photon waveform manipulation^17^,^21^,^22^,^23^, single photon quantum storage^24^ and imaging^25^.

We would like to start with the four-level double-Λ atomic system for biphoton generation, as shown in Figure 1(b). For the excited states |3〉 and |4〉 of ^85^Rb atoms, the population decay rates are Γ_3(4)_ = 2*π* × 6 MHz. The dephasing rates of the excited states are *γ*_13(14)_ = (1/2)Γ_3(4)_ for cold atomic ensemble. The states |1〉, |2〉 and 3〉 with a resonant strong coupling beam *ω_c_* constitute a EIT configuration. Though the transition between the state |1〉 and |2〉 is prohibited, the non-zero ground states dephasing rate *γ*_12_ is caused by atomic collisions, atomic thermal motions and Lamer precession of atoms in non-zero B field. In the MOT system we build, the Lamer precession of atoms are expected to be the main reason. Further we estimate the dephasing rate of the EIT coherence by measuring the probe beam transmission spectrum with the resonant coupling beam, and thus obtain *γ*_12_ = 2*π* × 0.03 MHz between 5*S*_1/2_*F* = 2 and 5*S*_1/2_*F* = 3. Figure 1(a) shows the backward geometry of four-wave mixing process, in which the opposite-arranged pump *ω_p_* and coupling beams *ω_c_* generate paired Stokes (*ω_s_*) and anti-Stokes (*ω_as_*) emitted in opposite directions. The biphotons, Stokes and anti-Stokes photons, are simultaneously generated from the SFWM nonlinear process in the atomic ensemble. The Stokes photons are usually far-detuned from atomic transitions, and therefore they propagate always with the speed of light in vacuum *c*. On the other hand, the anti-Stokes photons are produced with a resonant coupling laser beam, which creates a coherent EIT window for the photons to propagate through with a slow group velocity *V_g_*. In this regime, the EIT coherence determines the coherence of the paired photons. There are three important parameters to determine the slow light feature. The first one is the optical depth (OD) of the medium, defined as *OD* = *Nα*_0_*L*, where *N* denotes the atom density, *α*_0_ is the resonant absorption coefficient and *L* is the effective length of the atom cloud. The second parameter is the coupling field Rabi frequency Ω*_c_*, representing the coupling field strength. The last but not the least factor is the dephasing rate *γ*_12_ of the EIT coherence. When ignoring the dephasing rate, we could increase coherence time *T_c_* of the biphoton simply by prolonging the EIT delay time *τ_g_* = *L*/*V_g_*, which is proportional to the ratio *OD*/|Ω*_c_*|^2^. For example, Du *et al.*^26^ reported time-frequency entangled paired photons with 0.9 *μ*s coherence time, generated from a laser-cooled atomic cloud with optical depth (OD) of 53. From a dark-line two-dimensional MOT^29^ with OD as high as 130, Zhao *et al.*^27^ reported that the coherence time of these narrow-band entangled paired photons reaches 1.7 *μ*s. By phase locking two spatial symmetrical paths of the SFWM process, Liao *et al.*^28^ show polarization entangled narrow-band paired photon with the coherence time of 300 ns, which can be prolonged to 900 ns when they gradually reduce the coupling laser power. However, with a very low laser field coupling, the dephasing rate between two hyperfine states becomes important. Reducing the coupling laser power does not always prolong the coherence time *T_c_*, but instead results in a maximum *T_c_* at optimum Ω*_c_*. In the following sections, we demonstrate that the limit of *T_c_* is corporately determined by OD and the dephasing rate of the EIT ground-state coherence. In the case of extremely large OD, the coherence time of the biphoton finally approaches the ultimate limit determined by the dephasing rate. With an optimized dephasing rate and *OD* = 100 currently available in the system, we can realize a coherence time *T_c_* up to 2.34 *μ*s, which is the longest temporal length for biphoton reported to the best of our knowledge.

The two-photon state generated in the SFWM described in Figure 1 can be described by a temporal biphoton wavefunction^18^,
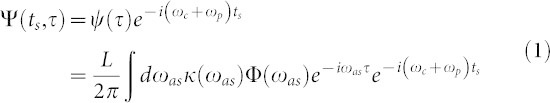
in which, *ψ* is the wavefunction amplitude, and *τ* = *t_as_* − *t_s_* denotes the delay time. 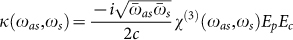 is the nonlinear parametric coupling coefficient and *χ*^(3)^ is the third-order nonlinear susceptibility. The longitudinal function 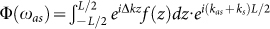, where Δ*k* = *k_as_* − *k_s_* − (*k_c_* − *k_p_*) cos *θ* is the phase mismatching for the backward geometry shown in Figure 1. Also, we include the intensity distribution *f*(*z*) of the pump or coupling beam along the *z* axis. According to experiment setup, we define a Gaussian beam distribution with a *e*^−2^ full width of *d* = 1.2 cm for both pump and coupling laser beams, and thus 
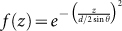
.

From equation(1), we obtain the numerical results for the joint-detection probability. Furthermore, equation(1) indicates that the spectrum of two-photon wavefunction is determined by the product of the *κ*(*ω*) and Φ(*ω*). Next we take some approximations to obtain an analytic solution for the wavefunction amplitude. Firstly, we assume that the EIT slow light effect play a dominant role to decide the two-photon wavefunction. Therefore we consider the case where Δ*ω_g_* < Ω*_e_*, in which 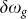 denotes the full width half maximum bandwidth of the function Φ(*ω*), and 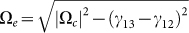 is the spectral separation of the two symmetric peak value of *κ*(*ω*)^26^. Now, Φ(*ω*) decides the two-photon wavefunction envelope, while 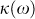 is approximated as a constant *κ*_0_. With this assumption, the coherence time of the paired photons is solely ascribed to the fact that, the anti-Stokes photons propagate through the atomic cloud with a slow velocity compared to the paired Stokes photons. Secondly, we assume *θ* → 0 or 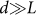 and thus *f*(*z*) ≈ 1 and the z integration term becomes a sinc function 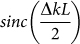. With the above assumption, we can simplify the wavefunction amplitude as,
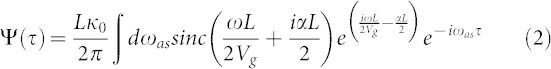
where, the EIT loss term 
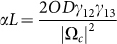
 is included. The EIT loss term *αL* is normally a small value close to 0. Through Taylor expansion at this small value, equation(2) gives an analytical solution after integral:
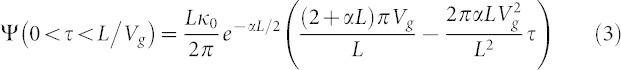
In other regions *τ* < 0 and *τ* > *L*/*V_g_*, Ψ(*τ*) = 0. If we ignore EIT loss term *αL* completely, equation(3) becomes a rectangular function Π(*τ*: 0, *τ_g_* = *L*/*V_g_*) as Refs. 18, and thus the 1/*e* coherence time is directly equals to EIT delay time *τ_g_*. Otherwise, the two-photon waveform amplitude exhibits a linear decay ending at *τ* = *τ_g_*(1/*αL* + 1/2). When *αL* tends to be infinity, the coherence time is bounded by *τ_g_*/2.

## Results

We prepare the cold atom cloud with optical depth *OD* = 100, and for SFWM process we set the coupling Rabi frequency as Ω*_c_* = 4.2*γ*_13_, 1.7*γ*_13_ and 1.2*γ*_13_, calculated from 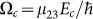. To guarantee the low gain limit condition for SFWM process, we use a low pump power corresponding to Ω*_p_* = 0.36*γ*_13_. Figure 2 shows the coincidence measurement results over these three cases, along with the numerical results of the two photon wavefunction amplitude equation(1). Figure 2(a) shows the case of Ω*_c_* = 4.2*γ*_13_. Apart from the biphoton optical precursor emerging immediately after *τ* = 0 ns, the two-photon joint detection waveform exhibits a Gaussian shape impressed from the controlling beams transverse beam profile *f*(*z*). At *τ* = 300 ns, the normalized cross-correlation function 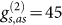. In contrast, we have 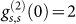 and 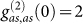. Thus the Cauchy-Schwartz inequality 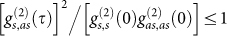 is violated by a factor of 506. Therefore, the generated photon pair source is proved to be nonclassical. Further, with the Stokes photon as reference, its paired anti-Stokes photon constitutes a heralded single photon. We measure the conditional second-order correlation function 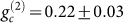 for window lengths of 500 ns, and hence we verify the Fock-state single photon nature of the heralded anti-Stokes photons. The 1/*e* coherence time of the two-photon waveform is 553 ns, and correspondingly the spectral bandwidth of the heralded single photon (also for the photon pair) is estimated as Δ*ω_g_* = 2*π* × 1.5 MHz, which is smaller than the atomic natural linewidth 2*π* × 6 MHz of ^85^Rb 5P state. Taking into account all the detection efficiencies and 10% of duty cycle, the photon pairs generated rate is 6436 pairs/s. Figure 2(b) shows the case of Ω*_c_* = 1.7*γ*_13_ with the same OD. The 1/*e* coherence time 2340 ns and corresponding bandwidth 2*π* × 0.38 MHz. The photon pair generation rate is 2043 pairs/s, with the maximum of the normalized cross-correlation function 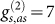. Therefore, the Cauchy-Schwartz inequality is still violated by a factor of 12. With a lower pump beam power Ω*_p_* = 0.21*γ*_13_, we obtain 
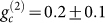
 for a 2400 ns coincidence window. Further decreasing the coupling power diminishes the visibility as Figure 2(c), Ω*_c_* = 1.2*γ*_13_, and distorts the two-photon waveform as a linear decay tail. The dash line in Figure 2(c) denotes the analytical solution of equation(3), and it matches the experimental data and numerical calculation well. The EIT loss term *αL* largely reduces the wavefunction visibility and maximum 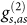 is around 3, with photon pair generation rate about 826 pair/s only. Considering the visibility, the near-rectangular waveform envelope in Figure 2(b) is the best we obtain with our present experimental setup.

The second important result is concerning the lower bound for coupling laser power. Figure 3 plots the measured 1/*e* coherence time (denoted as *T_c_* below) and 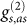 as a function of Ω*_c_* in four cases: *OD* = 22, 35, 50, 100. All of them satisfy the condition Δ*ω_g_* < Δ*ω_tr_*, in which 
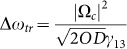
 denotes the spectral width of the EIT window. Accordingly, in Figure 3(a) we plot the numerical results simulated from equation(1), and for comparison, we also calculate the numerical curves for *OD* = 300, 1000. If we take the atomic ground states dephasing rate as *γ*_12_ = 2*π* × 45 kHz, the experimental data perfectly match the numerical curves, except that the data point at *OD* = 100, Ω*_c_* = 1*γ*_13_ is measured as 1900 ns, much lower than the calculated value. This is because the biphoton waveform visibility drops and some part of the coincidence counts are buried into the accidental counts. As is discussed above, at a low level of coupling field, the EIT loss term seriously damages the waveform shape, which instead exhibits a linear decay tail as equation(3). Therefore, Figure 3 shows that the numerical lines for various *OD*s all indicate a drop of *T_c_* at small Ω*_c_* and the optimum Ω*_c_* value increase with OD. On the other hand, Figure 3(b) shows the measured maximum of 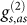 at these four OD cases. When Ω*_c_* < *γ*_13_, 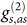 is below 2 and does not violate Cauchy-Schwartz inequality. The limit of the coherence time is corporately determined by *OD* and the atomic ground state dephasing rate *γ*_12_. Therefore, in Figure 4(a) we simulate the maximum *T_c_* as a function of OD when keeping *γ*_12_, and in Figure 4(b) vary *γ*_12_ when maintaining OD. Firstly, Figure 4(a) indicates extreme ODs bring the maximum *T_c_* to approach 3.5 *μ*s, which is determined by 1/*γ*_12_. *T_c_* glows very slow after *OD* > 200. On the other hand, Figure 4(b) shows a sensitive improvement of *T_c_* when reducing *γ*_12_. Hence, *γ*_12_ is the ultimate limiting factor. To further improve the biphoton coherence time, we need to minimize the dephasing rate *γ*_12_.

In conclusion, we have demonstrated that optical depth OD, the coupling field and the EIT dephasing rate corporately determine the biphoton coherence time. In a dense cold atom cloud, the biphoton coherence time is efficiently manipulated by the EIT slow light effect, and therefore can be increased with higher OD and lower coupling field amplitude. However, when the EIT coupling strength is reduced further, the dephasing rate *γ*_12_ becomes important and the EIT loss diminishes the two-photon waveform visibility, and thus ruin the nonclassical nature. The dephasing rate *γ*_12_ is the ultimate limiting factor. In a two-dimensional MOT system, *γ*_12_ is mainly caused by the quadruple field produced by the trapping coil. As in our experiment, through balancing the cooling beams power to position the atomic cloud along the field center, we can reduce *γ*_12_ to 283 kHz and thus produce paired photons with temporal coherence time 2.34 *μ*s with a reasonable OD of 100. The measured violation factor of the Cauchy-Schwartz inequality is 12. The conditional second-order auto-correlation function of anti-Stokes photons with reference to Stokes photons is measured as 
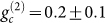
, which verifies the quantum Fock state nature. To push the limit even further, it is proved that shutting down the trapping magnetic field in the measurement window and maintaining a zero magnetic field at the atomic cloud is an efficient method to reduce *γ*_12_ to below 100 kHz^30^. According to our calculation shown in Figure 4(b), one could obtain *T_c_* = 10 *μ*s if *γ*_12_ = 0.004*γ*_13_ ≈ 75 kHz.

## Methods

**Figure 1:**
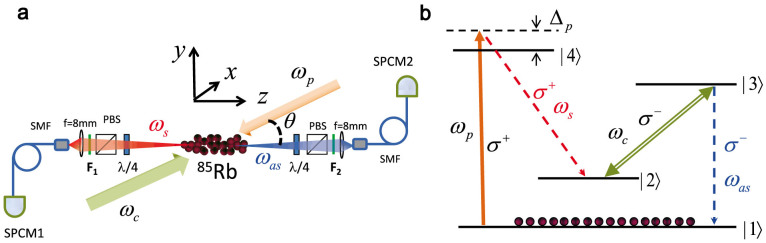
System schematics. (a), Backward SFWM experimental setup. The ^85^Rb atomic ensemble is prepared in a two-dimensional magneto-optical trap. (b), Energy level scheme for SFWM. The four energy levels activated are: |1〉 = 5*S*_1/2_*F* = 2 (initial atomic state), |2〉 = 5*S*_1/2_*F* = 3, |3〉 = 5*P*_1/2_*F* = 3 and |4〉 = 5*P*_3/2_*F* = 3.

**Figure 2:**
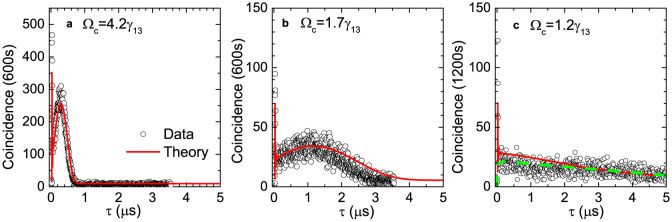
Accumulated coincidence counts measurements. (a), Ω*_c_* = 4.2*γ*_13_ (over 600 s); (b), Ω*_c_* = 1.7*γ*_13_ (over 600 s); (c) Ω*_c_* = 1.2*γ*_13_ (over 1200 s). black circles denote the experimental data, and the red solid curve denotes the numerical results calculated from Eq. 1. The green dash line denotes the equation (3). Ω*_p_* = 0.36*γ*_13_, *α*_0_*L* = 100.

**Figure 3:**
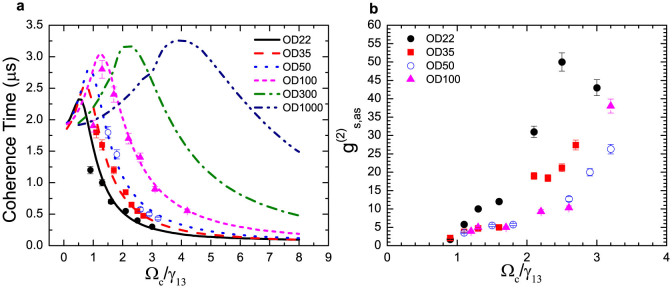
Experimental and theoretical results when varying Ω*_c_*. (a), Paired photons coherence time as a function of coupling Rabi frequency Ω*_c_*. The cold atom cloud is prepared with various OD conditions, and the curves represent the numerical results. The black filled circles, red squares, blue empty circles and purple triangles denote experimental data for *OD* = 22, 35, 50 and 100, respectively. (b), The experimental data of the maximum 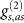.

**Figure 4:**
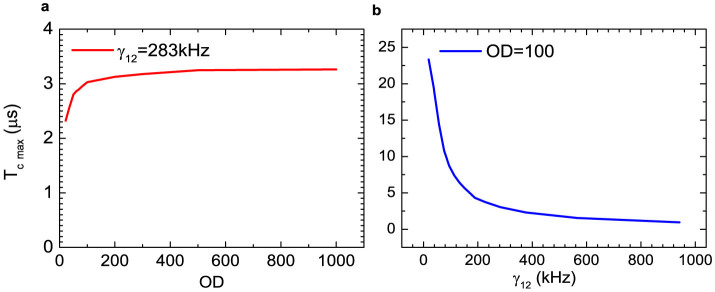
Coherence time limit. (a), *T_c_* limits as a function of OD, with *γ*_12_ = 0.015*γ*_13_ = 283 kHz. (b), *T_c_* limits as a function of *γ*_12_, with *OD* = 100.

The cold atomic ensemble is prepared in a two-dimensional ^85^Rb magneto-optical trap (MOT) with length *L* = 1.5 cm. The cooling laser beam is red-detuned from the atomic transition 5*S*_1/2_*F* = 3 → 5*P*_3/2_*F* = 4 by 20 MHz. The total power of the six cooling beams is 130 mW, and each beam has an intensity of 6.42 *mW*/*cm*^2^. The repump beam is on-resonance to the atomic transition 5*S*_1/2_*F* = 2 → 5*P*_3/2_*F* = 2, which collects the atoms back to the cooling circulation. The total power of the repump beam is 30 mW, and it is applied onto the MOT along ±*x* axis shown in Figure 1. The system is run periodically with a MOT preparation time of 4.5 ms followed with a measurement window of 0.5 ms. The repump beam switches off 300 *μs* before the cooling beam is off, and therefore over 99% of trapped atoms are pumped to the ground state 5*S*_1/2_*F* = 2. The center gradient of the 2D MOT quadrature magnetic field is 10 G/cm and remains always on. To minimize the Zeeman shift for atomic energy state, we carefully balance the six cooling beams to align the laser cooled atoms along the positions of zero transverse B field.

After the atoms are cooled and trapped, we apply continuous pump (*ω_p_*) and coupling (*ω_c_*) lasers within the measurement window. Within the measurement window, the pump and coupling beam are present simultaneously on the cold atom cloud, with polarization *σ*^+^ and *σ*^−^ respectively. The paired Stokes (*ω_s_*) with polarization *σ*^+^ and anti-Stokes (*ω_as_*) photons *σ*^−^ are emitted in opposite directions, to satisfy the angular momentum conservation in the FWM process. We collect the paired photons along the longitudinal axis (*z* axis as denoted), along which we obtain the largest OD for the cloud. To minimize the scattered optical noise from the strong laser beams, the pump-coupling axis is deviated from the *z* axis by *θ* = 3°. The pump beam is blue-detuned to the transition |1〉 → |4〉 by Δ*_p_* = 146 MHz, to avoid strong excitation and maintain low gain limit. The coupling beam is on-resonance to the transition |2〉 → |3〉, and therefore constitute a three-level Λ EIT scheme for anti-Stokes photons.

Either of the collection end is composed of a quarter wave plate followed by a polarization beam-splitter, a single mode fiber (SMF, fiber-fiber coupling efficiency 70%), and a Fabry-Perot filter (each with 70% transmission), and finally a single photon counting module (SPCM-AQRH-14-FC from Excelitas, each with 50% detection efficiency). The coincidence measurements are carried out with Time to Digital Converter (DPC-230 from Becker & Hickl GmbH).

## Author Contributions

J.F.C. designed the experiment. Z.H. and P.Q. built up the system. Z.H. performed the experiment and analyzed the data. L.Z. worked out the analytical solution. J.F.C. wrote the manuscript and all authors contributed to the final manuscript. The whole program is under the supervision of W.P.Z.
